# DeltaNeTS+: elucidating the mechanism of drugs and diseases using gene expression and transcriptional regulatory networks

**DOI:** 10.1186/s12859-021-04046-2

**Published:** 2021-03-04

**Authors:** Heeju Noh, Ziyi Hua, Panagiotis Chrysinas, Jason E. Shoemaker, Rudiyanto Gunawan

**Affiliations:** 1 Institute for Chemical and Bioengineering, ETH Zurich, 8093 Zurich, Switzerland; 2 Swiss Institute of Bioinformatics, 1015 Lausanne, Switzerland; 3 Department of Chemical and Biological Engineering, University at Buffalo – SUNY, Buffalo, NY 14260 USA; 4 Department of Chemical and Petroleum Engineering, University of Pittsburgh, Pittsburgh, PA 15261 USA; 5 Department of Computational and Systems Biology, University of Pittsburgh, Pittsburgh, PA 15261 USA; 6 Present Address: Columbia University Medical Center, New York, NY 10032 USA

**Keywords:** Gene expression, Gene regulatory network, Gene targets, Drug discovery, Mechanism of action

## Abstract

**Background:**

Knowledge on the molecular targets of diseases and drugs is crucial for elucidating disease pathogenesis and mechanism of action of drugs, and for driving drug discovery and treatment formulation. In this regard, high-throughput gene transcriptional profiling has become a leading technology, generating whole-genome data on the transcriptional alterations caused by diseases or drug compounds. However, identifying direct gene targets, especially in the background of indirect (downstream) effects, based on differential gene expressions is difficult due to the complexity of gene regulatory network governing the gene transcriptional processes.

**Results:**

In this work, we developed a network analysis method, called DeltaNeTS+, for inferring direct gene targets of drugs and diseases from gene transcriptional profiles. DeltaNeTS+ uses a gene regulatory network model to identify direct perturbations to the transcription of genes using gene expression data. Importantly, DeltaNeTS+ is able to combine both steady-state and time-course expression profiles, as well as leverage information on the gene network structure. We demonstrated the power of DeltaNeTS+ in predicting gene targets using gene expression data in complex organisms, including *Caenorhabditis elegans* and human cell lines (T-cell and Calu-3). More specifically, in an application to time-course gene expression profiles of influenza A H1N1 (swine flu) and H5N1 (avian flu) infection, DeltaNeTS+ shed light on the key differences of dynamic cellular perturbations caused by the two influenza strains.

**Conclusion:**

DeltaNeTS+ is a powerful network analysis tool for inferring gene targets from gene expression profiles. As demonstrated in the case studies, by incorporating available information on gene network structure, DeltaNeTS+ produces accurate predictions of direct gene targets from a small sample size (~ 10 s). Integrating static and dynamic expression data with transcriptional network structure extracted from genomic information, as enabled by DeltaNeTS+, is crucial toward personalized medicine, where treatments can be tailored to individual patients. DeltaNeTS+ can be freely downloaded from http://www.github.com/cabsel/deltanetsplus.

## Background

Analyzing the molecular mechanism of drugs and diseases is a central task in drug discovery. For drugs, this task involves identifying the molecular targets whose interaction with the compound is associated with its pharmacological activity. The knowledge of the molecular mechanism of action (MOA) of a drug is important in ascertaining not only the therapeutic efficacy of this drug, but also any potential toxicity and side effects. On the other hand, insights into the molecular mechanism of a disease may lead to a better understanding of its pathogenesis and possible to new and better treatment formulations. In this regard, gene transcriptional profiling has emerged as a viable high-throughput platform for drug discovery and drug target identification [1, 2] and for studying disease mechanism [3]. However, determining the direct molecular targets through which a drug or a disease exert its effects using gene transcriptional profiles remains a major bioinformatics challenge. Changes in the gene expression caused by a drug or a disease may arise directly from the action of the drug or disease, or indirectly as downstream or secondary effects. Delineating direct and indirect gene targets from gene transcriptional profiles is further complicated by the fact that gene expression is a highly regulated process that involves a complex and context-specific gene regulatory network (GRN).

Many strategies have been proposed for inferring gene targets from gene transcriptional data [4]. In general, these strategies fall in two main categories: comparative analysis and network analysis methods. The former involves comparing the transcriptional profiles of interest with a library of reference profiles with known targets [1, 2, 5, 6], under the assumption that a likeness in the transcriptional profiles is indicative of similarity in the molecular targets. The latter class of methods uses a model of GRN to account for gene transcriptional regulations when analyzing gene expression data. A number of network analysis methods, notably causal analysis [7, 8] and DeMAND [9], employs graph models of the GRNs with nodes representing the genes and edges representing gene–gene interactions. Here, gene target identification is typically formulated as a statistical hypothesis test using the gene transcriptional profiles. Another group of network analysis methods, including network identification by multiple regression (NIR) [10], mode of action by network identification (MNI) [11], sparse simultaneous equation model (SSEM) [12], and DeltaNet [13], relies on a mechanistic model of the gene transcriptional regulatory network. By invoking a pseudo-steady-state assumption, the inference of gene targets from gene expression data is recasted as a regression problem. Generally speaking, a gene is scored highly as a potential target when its expression deviates significantly from what the GRN model predicts based on the expression of its transcription factors (TFs).

Here, we present a much-improved network analysis method of our previous algorithm DeltaNet [13] to address two shortcomings: analysis of time-series data and incorporation of prior information on the GRN structure. As we have demonstrated earlier [14], DeltaNet performed poorly when using time-series transcriptional data due to the invalidity of the underlying steady-state assumption. Such an issue likely applies to similar algorithms employing pseudo steady-state assumption, such as NIR, MNI and SSEM. There is a clear need to accommodate time-series expression data, as they constitute an important class of gene expression datasets. A notable example is the Connectivity Map (CMap), which includes over 1.5 million time-series gene expression profiles from 9 different cell types across roughly 5000 small-molecule compounds and 3000 genetic reagents.

The second shortcoming of any network analysis methods is the uncertainty in the gene network model employed in the analysis. In DeltaNet, as well as in NIR, MNI and SSEM, the GRN is reconstructed from the gene expression data as an intermediate step or implicitly in the inference. But, as we and many others have shown [15, 16], such GRN reconstructions constitute an undetermined problem and thus the reconstructed GRN often has high uncertainty. In parallel, we have seen a tremendous progress over the past decade in the mapping of transcriptional regulatory elements [17], the measurements of promoter/enhancer activity [18], and the identification of TF binding motifs and binding sites [19]. Such information has enabled the reconstruction of GRN graphs for ~ 300 cell types in human [20]. Thus, there is an obvious opportunity and necessity to combine diverse datasets on the GRN structure with the gene transcriptional data within the network analysis paradigm for gene target inference.

In this work, we developed DeltaNeTS+. DeltaNeTS+ is capable of combining steady-state and time-series gene transcriptional data for gene target scoring. DeltaNeTS+ is further able to incorporate prior information of the structure of the GRN for the target inference. We demonstrated the superiority of DeltaNeTS+ over well-established procedures, including TSNI (Time Series Network Identification) [21], DeMAND [9], and differential expression analysis, using gene transcriptional datasets of complex organisms, including *C. elegans* and human. Notably, the application of DeltaNeTS+  to gene expression datasets from human lung Calu-3 cells infected by influenza strains H5N1 (avian flu) and H1N1 (swine flu) reveals key differences in the cellular responses to the two strains.

## Methods

### DeltaNeTS+

DeltaNeTS+ is a network analysis method for inferring causal gene targets from time series gene expression data. DeltaNeTS+ relies on an ordinary differential equation (ODE) model of gene transcriptional process, as follows [22]:

1 $$\frac{{dr_{k} }}{dt} = u_{k} \mathop \prod \limits_{{\begin{array}{*{20}c} {j = 1} \\ {j \ne k} \\ \end{array} }}^{n} r_{j}^{{a_{kj} }} - d_{k} r_{k}$$

where *r*_*k*_ denotes the concentration of gene *k* transcripts (mRNA), *u*_*k*_ and *d*_*k*_ denote the transcription and degradation rate constants of gene *k*, respectively, *a*_*kj*_ denotes the regulatory control of gene *j* on gene *k*, and *n* is the number of genes. The magnitude and sign of *a*_*kj*_ indicate the strength and mode of the regulatory interaction, respectively. A positive *a*_*kj*_ indicates activation, while a negative *a*_*kj*_ indicates repression. As commonly done, *a*_*jj*_’s are assumed to be zero (i.e., no self-regulatory loops). Taking pseudo steady-state assumption and logarithm transformation, Eq. (1) can be simplified into,

2 $$c_{ki} = \mathop \sum \limits_{j = 1}^{n} a_{kj} c_{ji} + p_{ki}$$

where *c*_*ki*_ = log_2_(*r*_*ki*_/*r*_*kb*_) denotes the log-twofold change (log2FC) of mRNA levels of gene *k* between treatment sample *i* and corresponding control *b*, and *p*_*ki*_ = log((*u*_*ki*_/*d*_*ki*_) / (*u*_*kb*_ / *d*_*kb*_)) denotes the effects of treatment in sample *i* on gene *k*. The variable *p*_*ki*_ is the unknown variable of interest. The formulation in Eq. (2) shows that the expression change of gene *k* by the treatment arises from the indirect effects through its transcriptional regulators (the summation in Eq. (2)) and the direct effect (the last term in Eq. (2)). More specifically, the variable *p*_*ki*_ gives the amount of fold-change expression of gene *k* that cannot be explained by the fold-change expression of its transcriptional regulators. Here, we use *p*_*ki*_ as a measure of the direct perturbations on gene *k* by the treatment. A positive (negative) *p*_*ki*_ indicates that the treatment causes a higher (lower) level of gene *k* transcription than what is expected from the changes in the transcription factors or regulators of the gene. In general, the larger the magnitude of *p*_*ki*_, the stronger is the perturbations to gene *k* in the sample *i.*

As we often have more than one samples for a given treatment (e.g., technical repeats, multiple time points), we may assign the same perturbations to all of the related samples. For *M* different treatments among *m* samples (*M* < *m*), the following equation in a matrix–vector format can be considered:

3 $${\mathbf{C}} = {\mathbf{AC}} + {\mathbf{PZ}}$$

where **C** is the *n* × *m* matrix of log2FCs of *n* genes in *m* samples, **A** is the *n* × *n* matrix of *a*_*kj*_’s describing the GRN, **P** is the *n* × *M* matrix of perturbation coefficients, and **Z** is an *M* × *m* {0, 1} mapping matrix that assigns the specific perturbation variable *p* to the appropriate sample. Note that **Z** becomes an *m* × *m* identity matrix when the perturbations are treated as distinct among the entire *m* samples.

For time-series data, we assume that mRNA concentrations vary between sampling time points such that

4 $${\text{log}}\left( {r_{k}^{l + 1} } \right) = s_{k}^{l} t + {\text{log}}\left( {r_{k}^{l} } \right)\;{\text{for}}\;t_{l} \le t \le t_{l + 1}$$

where $t_{l}$ denotes the *l-*th time point, $r_{k}^{l}$ is the mRNA concentration of gene *k* at the *l*-th time point, and $s_{k}^{l}$ is the slope between the two time points. Using Eq. (1), the first order derivative of the logarithm of *r*_*k*_ can be rewritten as the following:

5 $$\frac{{d{\text{log}}\left( {r_{k} } \right)}}{dt} = \frac{{u_{k} }}{{r_{k} }}\mathop \prod \limits_{{\begin{array}{*{20}c} {j = 1} \\ {j \ne k} \\ \end{array} }}^{n} r_{j}^{{a_{kj} }} - d_{k} = u_{k} \exp \left( {\mathop \sum \limits_{{\begin{array}{*{20}c} {j = 1} \\ {j \ne k} \\ \end{array} }}^{n} a_{kj} \log \left( {r_{j} } \right) - \log \left( {r_{k} } \right)} \right) - d_{k}$$

By substituting Eq. (4) for *r*_*k*_ in Eq. (5), the following equation can be derived for $t_{l} \le t \le t_{l + 1}$:

6 $$\frac{{d{\text{log}}\left( {r_{k} } \right)}}{dt} = u_{k} \exp \left( {\mathop \sum \limits_{{\begin{array}{*{20}c} {j = 1} \\ {j \ne k} \\ \end{array} }}^{n} a_{kj} \left( {s_{j}^{l} t + {\text{log}}\left( {r_{j}^{l} } \right)} \right) - \left( {s_{k}^{l} t + {\text{log}}\left( {r_{k}^{l} } \right)} \right)} \right) - d_{k}$$

The derivative of Eq. (6) (i.e. the second derivative of log(*r*_*k*_)) is zero under the pseudo steady-state assumption made in Eq. (4).

7 $$\frac{{d^{2} {\text{log}}\left( {r_{k} } \right)}}{{dt^{2} }} = 0 = u_{k} \exp \left( {\mathop \sum \limits_{{\begin{array}{*{20}c} {j = 1} \\ {j \ne k} \\ \end{array} }}^{n} a_{kj} \left( {s_{j}^{l} t + {\text{log}}\left( {r_{j}^{l} } \right)} \right) - \left( {s_{k}^{l} t + {\text{log}}\left( {r_{k}^{l} } \right)} \right)} \right)\left( {\mathop \sum \limits_{{\begin{array}{*{20}c} {j = 1} \\ {j \ne k} \\ \end{array} }}^{n} a_{kj} s_{j}^{l} - s_{k}^{l} } \right)$$

Here, *u*_*k*_ and *d*_*k*_ are assumed to be the same between the *l*-th and *l* + 1-th time points, i.e. the perturbations are constant in this time window. Since *u*_*k*_ and the exponential term in Eq. (7) are always positive, the remaining term should be zero. Therefore, the following relationship between the variable $s_{k}^{l}$ and the network coefficient *a*_*kj*_ can be obtained:

8 $$s_{k}^{l} = \mathop \sum \limits_{{\begin{array}{*{20}c} {j = 1} \\ {j \ne k} \\ \end{array} }}^{n} a_{kj} s_{j}^{l}$$

The matrix form of Eq. (8) for *n* genes and *m* samples is the same as following:

9 $${\mathbf{S}} = {\mathbf{AS}}$$

where **S** is the *n* × *m* matrix of the slopes calculated from time series log2FCs data. In DeltaNeTS+, the slopes of the time series gene expression profiles were calculated using 2nd-order accurate finite difference approximations at each sampling time point [23]. For the first and last time points, forward and backward finite difference were used, respectively, while for middle time points, a centered difference approximation was used. At the minimum, only two time points are needed to compute the time slopes, but having more time points enables implementing finite differencing with a higher order of accuracy.

Combining Eq. (3) and Eq. (9), DeltaNeTS+ calculates the unknown variables **A** and **P** row by row using the following equation:

10 $$\left[ {\begin{array}{*{20}c} {{\overline{\mathbf{C}}}_{k}^{{\text{T}}} } \\ {{\overline{\mathbf{S}}}_{k}^{{\text{T}}} } \\ \end{array} } \right] = \left[ {\begin{array}{*{20}c} {{\overline{\mathbf{C}}}^{{\text{T}}} } & {\mathbf{Z}} \\ {{\overline{\mathbf{S}}}^{{\text{T}}} } & {\mathbf{0}} \\ \end{array} } \right]\left[ {\begin{array}{*{20}c} {{\mathbf{A}}_{k}^{{\text{T}}} } \\ {{\mathbf{P}}_{k}^{{\text{T}}} } \\ \end{array} } \right]$$

where ${\overline{\mathbf{C}}}_{k}^{{}}$, ${\overline{\mathbf{S}}}_{k}^{{}}$, **A**_*k*_, and **P**_*k*_ are the row vectors of the matrices ${\overline{\mathbf{C}}}$, ${\overline{\mathbf{S}}}$, **A**, and **P** for gene *k* and **0** is the *m* × *M* zero matrix. The matrices ${\overline{\mathbf{C}}}$ and ${\overline{\mathbf{S}}}$ are the normalized log2FC and slope matrices ${\mathbf{C}}$ and ${\mathbf{S}}$ such that each of the matrices has a 2-norm equal to the square root of the number of samples in the matrix (i.e. the number of columns in the matrix). The normalization is set to balance the contributions from the two matrices in determining **A** and **P**.

In DeltaNeTS+, when information on the structure of the GRN is available—for each gene *k*, we have information on its transcription factors—we can reduce the dimension of the problem stated in Eq. (10) by restricting the inference of **A** only for the subset of genes related to the TFs of gene *k*:

11 $$\left[ {\begin{array}{*{20}c} {{\overline{\mathbf{C}}}_{k}^{{\text{T}}} } \\ {{\overline{\mathbf{S}}}_{k}^{{\text{T}}} } \\ \end{array} } \right] = \left[ {\begin{array}{*{20}c} {{\overline{\mathbf{C}}}_{{{\text{TF}}_{k} }}^{{\text{T}}} } & {\mathbf{Z}} \\ {{\overline{\mathbf{S}}}_{{{\text{TF}}_{k} }}^{{\text{T}}} } & {\mathbf{0}} \\ \end{array} } \right]\left[ {\begin{array}{*{20}c} {{\mathbf{A}}_{{{\text{TF}}_{k} }}^{{\text{T}}} } \\ {{\mathbf{P}}_{k}^{{\text{T}}} } \\ \end{array} } \right]$$

where the subscript TF_*k*_ refers to the subset of genes corresponding to the TFs of gene *k*. Equation (11) is solved using ridge regression to give ${\mathbf{A}}_{{{\text{TF}}_{k} }}^{{}}$ and ${\mathbf{P}}_{k}^{{}}$ matrices for the entire GRN [24]. When GRN structure is not provided or not available, DeltaNeTS+ solves Eq. (10) by using LASSO regularization to predict a sparse matrix **A**_*k*_ and **P**_*k*_ [14]. We implemented LASSO [25] and ridge regression in DeltaNeTS+ using the GLMNET algorithm with *k-*fold cross-validation (default *k* = 10) [26]. Consequently, the smallest number of samples that DeltaNeTS+ can handle is *k* samples.

### Gene expression data

We applied DeltaNeTS+ to time-series gene expression data of *C. elegans* embryo [27], human cord blood CD4+T cells [28], and human lung cancer Calu-3 cells [29–31]. For *C. elegans* embryo, log2 intensity data which were normalized by robust multi-array average (RMA) method were obtained from Gene Expression Omnibus (GEO) [32] (accession number: GSE2180 and GSE51162). Log2FC of gene expressions and its statistical significance (Benjamini–Hochberg adjusted *p *value) between gene knockout and wild-type conditions at each time point were calculated using a linear fit model and empirical Bayes method in the *limma* package of Bioconductor. The probe sets were mapped to the official gene symbols in *celegans.db*. In the case of multiple probe sets mapping to a gene symbol, we take the log2FC from the probe set with the smallest average adjusted *p* value over the samples.

For human T cells, log2 intensity data by quantile normalization were obtained from GEO (accession number: GSE17851). In the same way as *C. elegans*, log2FC values were calculated between gene knockout and wild-type conditions at each time point using *limma*. The probe sets were mapped to the gene symbols from the Illumina human-6 v2.0 expression beadchip data in GEO (accession number: GPL6102), and for the multiple probe sets mapping to the same gene, the probe set with the smallest average adjusted *p* value across all samples was chosen.

For Calu-3 data, we compiled the raw *Agilent Whole Human Genome 4* × *44 K* microarray data from GSE33264 for IFN-α and IFN-γ experiments [31] and from GSE37571 and GSE33142 for H1N1 and H5N1 experiments [29, 30]. The raw data were background-corrected and normalized using *normexp* and *quantile* methods in *limma* package of Bioconductor. The log2FCs between virus (or interferon) and mock samples at each time point were calculated using *limma*, with their statistical *p* value adjusted by Benjamini-Hockberg method. The probe sets were mapped to the official gene symbols in *hgug4112a.db* package. For a gene with multiple probe sets, we chose the data from the probe set with the smallest average adjusted *p* value.

During preprocessing of time-series data, we substituted any time-series log2FC gene expression data that were not statistically significant with linearly interpolated values using adjacent time points with statistically significant log2FCs. Note that the computation of statistical significance (*p *value) requires at least three repeats, and thus, the linear interpolation using adjacent time points above is done only when 3 or more repeats are available. Unless mentioned differently, we used Benjamini–Hochberg adjusted *p* value < 0.05 to establish statistical significance. If the log2FC values of a gene were not statistically significant at any time point, we set the log2FCs to zero.

### Gene regulatory networks

The GRN for *C. elegans* embryo data set was obtained from TF-gene interactions for *C. elegans* in TF2DNA database [33], where TF binding motifs and their regulated genes were identified by calculating the binding affinity based on the known 3D structure of TF-DNA complexes. The GRN for *C. elegans* was composed of 355,080 edges from 48 TFs to 15,738 genes.

Meanwhile, the GRNs for human T-cell and Calu-3 data sets were obtained from TF-gene interactions specific for human cord blood-derived cells and human epithelium lung cancer cells, respectively, available in Regulatory Circuit database [20]. We only used TF-gene interactions with a confidence score greater than 0.1 in the Regulatory Circuit database. The GRNs for human T-cells and Calu-3 consisted of 11,955 edges pointing from 438 TFs to 2,385 genes and 42,145 edges pointing from 515 TFs to 7,125 genes, respectively.

### TSNI and DeMAND implementation

For TSNI [21], we downloaded the MATLAB subroutine from https://dibernardo.tigem.it/softwares/time-series-network-identification-tsni and applied it to *C. elegans*, human T-cell, and Calu-3 interferon data. The parameter of the number of principle components in TSNI was optimized to nPC = 1—i.e. using only the first principal component. For DeMAND [9], we installed the DeMAND R-package from Bioconductor [34] (https://www.bioconductor.org) and applied the method using the same GRNs used in DeltaNeTS+ analysis.

### Enrichment analysis of Calu-3 data

For interferon case study, the top 50 genes ranked by each method (DeltaNeTS+, TSNI, and log2FC) were used for Gene Ontology (GO) and Reactome pathway enrichment analysis using Enrichr [35]. For influenza A viral infection case study, the averaged **P** values of DeltaNeTS+ for each phase (phase 1: 0 to 7 h, phase 2: 7 to 18 h, phase 3: > 18 h) were used for the gene set enrichment analysis (GSEA) of Reactome pathways [36] using ReactomePA package [37] in R. Before the enrichment analysis, genes were sorted based on the average **P** for each time phase, and genes with no perturbation score were excluded during the GSEA. Afterwards, the significance score (− log_10_ *p* value) of the enriched pathways was calculated among the pathways with positive enrichment score from GSEA. The illustration of the results in Fig. 2 only shows the highest level of pathway information in the Reactome hierarchy (https://reactome.org/PathwayBrowser), while the significance score is the best significance score (highest − log_10_ *p* value) of the sub-pathways.

### Weighted gene co-expression network analysis (WGCNA)

WGCNA [38] was applied to the DeltaNeTS+ score of both H1N1 (influenza A/CA/04/09) and H5N1 (influenza A/VN/1203/04) samples. The H1N1 data were from 9 time points (0, 3, 7, 12, 18, 24, 30, 36, and 48 h) and the H5N1 data were from 6 time points (0, 3, 7, 12, 18, and 24 h). In WGCNA analysis, we computed modules with a minimum of 200 genes using the signed network option (soft-thresholding power = 18). Afterwards, GO and Reactome enrichment analysis were also performed for each module using ReactomePA and clusterProfiler packages in R.

## Results

### Predicting genetic perturbations

We tested the performance of DeltaNeTS+ by inferring gene perturbations from time series *C. elegans* and human T-cell gene expression profiles from RNA interference (RNAi) experiments. DeltaNeTS+ generates gene perturbation scores *p*_*ki*_ for all genes using the entire GRN. *p*_*ki*_ indicates the strength of perturbations to the expression of gene *k* in sample *i*. In the following, we used the magnitude of the perturbation scores to rank the gene targets predicted by DeltaNeTS+. The *C. elegans* dataset comprises three genetic perturbation experiments [27], each of which provides gene expression data across 10 time points after *skn*-1 and *pal*-1 knockdowns by shRNA on *mex*-3 or *pie*-1 mutated cells. The human T-cell dataset includes time-series gene expression data measured over 4 time points after STAT6 knockdown by shRNA [28] (see Table 1). When applying DeltaNeTS+ on these data, gene regulatory network graphs for *C. elegans* and human T-cells were used as prior information (see Methods).

**Table 1:** Rank prediction of gene targets in *C. elegans* and human T-cell data sets by DeltaNeTS+, TSNI, DeMAND, and log2FC magnitudes. The ranking is computed based on the magnitude of target scores for all genes in the GRN from each algorithm (see Methods)

	Experiment 1		Experiment 2		Experiment 3
*C. elegans* data set					
Methods	mex-3	skn-1	pie-1	pal-1	pie-1
DeltaNeTS+	9	44	9	2	2
TSNI	11	5086	13	7	13
DeMAND	38	15,150	23	3	38
log2FC	3	8623	4	304	3

	Experiment 1	Experiment 2
Human T-cell data set		
Methods	STAT6	STAT6
DeltaNeTS+	1	1
TSNI	1	22
DeMAND	1	2
log2FC	1	4

For comparison purposes, we generated gene target predictions using TSNI [21] and DeMAND [9] and those based on the magnitudes of log2FC values. For log2FC analysis, the gene target candidates were ranked in decreasing order of the absolute value of the log2FCs, while for DeMAND, the genes were ranked in increasing order of the statistical *p* value of dysregulation [9]. For TSNI, we used the first principal component to generate the gene target predictions as this setting gave the best accuracy among the trials using principle components between 1 and 3. We also compared the accuracy of the gene target predictions under two different scenarios; the first is where the gene perturbations are time invariant, and the other is where the gene perturbations are allowed to vary over time. Because of the small number of samples in the above datasets, when the GRN structure is not used, the application of DeltaNeTS+ using LASSO (see Methods) and a previous algorithm DeltaNet resulted in an empty target prediction, where the perturbation scores **P** were 0.

Table 1 shows the ranking of the genes targeted by shRNA or mutation in *C. elegans* and human T-cell data with the gene perturbations set to be time-invariant, i.e. the gene perturbations are the same for all samples from the same treatment/condition. Except for *skn*-1, DeltaNeTS+ placed the known targets (*mex*-3, *pal*-1, *pie*-1, and STAT6) among the top 10 of the candidate gene targets. Notably, in almost all instances, DeltaNeTS+ ranked the known targets higher than TSNI, DeMAND, and log2FC analysis. The transcription factor *skn*-1 regulates critical biological pathways related to oxidative stress responses and lifespan of *C. elegans* [39]. Silencing *skn*-1 alters the transcription of a large number of genes (> 10,000), which complicates the identification of the true gene perturbation in these samples. Here, a significant fraction of gene targets predicted by DeltaNeTS+ with higher ranks than *skn-*1 were genes regulated by *skn*-1 (see Additional file 1). Nevertheless, DeltaNeTS+ was able to put *skn*-1 at a much higher rank than TSNI, DeMAND, and log2FC.

**Fig. 1:**
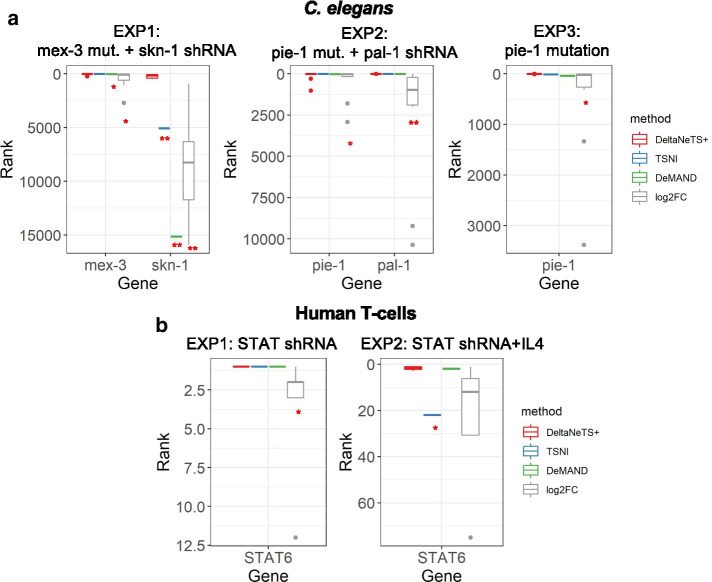
Ranks predictions for knock-out targets. The distribution of box plot indicates the ranks from all the time points from the same experiment. The ranks of gene targets by DeltaNeTS+ are colored in red and those by TSNI, DeMAND, and log2FC analysis are colored in blue, green, and grey, respectively. *p value < 0.1 and **p value < 0.01 by Wilcox signed rank test between DeltaNeTS and other methods

When using time-varying perturbation (i.e. the targets can vary across different time samples of the same treatment/condition), DeltaNeTS+ again performed better than the three methods TSNI, DeMAND, and log2FC, as illustrated in Fig. 1. The log2FC of samples from the early time points actually gave a reasonably accurate indication of the direct gene perturbations (see Additional file 2: Tables S1–S2). But, log2FC magnitude became drastically a less accurate indicator for the direct gene targets for the later time points. This trend is expected because the downstream effects of a gene perturbation will progressively mask the true gene target identity over time. In comparison, DeltaNeTS+ prediction accuracy degraded much more mildly over the sampling times, demonstrating its higher robustness with respect to the choice of time samples (see Additional file 2: Tables S1–S2). Note that TSNI and DeMAND only provide an overall target prediction, and not for different time points.

### Predicting the mechanism of interferon-α and interferon-γ actions

Next, we tested the ability of DeltaNeTS+ in differentiating the specific activity of two related compounds from the same family of proteins: interferon-α (IFN-α) and interferon- γ (IFN-γ). This task is challenging as these two signalling proteins trigger a common response of the immune system but through different signalling pathways. IFN-α and IFN-γ are cytokines that induce innate immune response against viral infection through separate signalling pathways, called type I and type II signalling, respectively. The IFN-α signal goes through type I IFN receptor and is followed by the formation of the complex IRF9, STAT1 and STAT2 that activates the transcription of IFN-α/β stimulated genes (ISG). Meanwhile, the IFN-γ signal is received by type II IFN receptor which leads to the transcription of genes containing gamma interferon activation sites (GAS) [40, 41]. The gene expression data came from a previous study using human lung cancer cells Calu-3 [31]. We employed the human epithelial lung cancer cell GRN structure as prior information for DeltaNeTS+ (see Methods). To examine cellular pathways that are perturbed by each of the two interferon treatments, we performed GO (Biological Processes) and Reactome pathway enrichment analysis for the 50 highest ranked genes from each method [35].

Table 2 summarizes the enriched GO terms and Reactome pathways of the top gene target predictions from DeltaNeTS+, DeMAND and log2FC analysis (adjusted *p* value < 0.01; detailed results in Additional file 3). The top 50 gene targets predicted by TSNI did not show any statistically significant enrichment for neither GO nor Reactome pathways. The enriched GO and Reactome terms of the DeltaNeTS+ perturbation analysis correctly point to the distinct signalling pathways through which the two interferons act. Besides interferon-specific signalling, pathways related to major histocompatibility complex (MHC) class II antigen, which is induced by an IFN-γ stimulated gene CIITA [42], are also enriched in the DeltaNeTS+ analysis for IFN-γ data, but not for IFN-α data. On the other hand, the enrichment analysis of top log2FC genes shows more diverse over-represented pathways, which is expected as the log2FC expressions reflect both direct and indirect effects of the two interferons. While the distinct signalling pathways related to IFN-α and IFN-γ are among the top enriched GO and Reactome pathways for log2FC, the signalling pathways specific to each interferon are cross-listed in the enrichment results for the two interferons. For DeMAND, no interferon signalling was detected for IFN-γ data although type I and II interferon signalling pathways were enriched from the DeMAND prediction for IFN-α data. In comparison to DeltaNeTS+, log2FC and DeMAND were less capable in differentiating the specific signalling pathways through which IFN-α and IFN-γ act.

**Table 2:** Reactome pathway enrichment analysis for Interferon-α and -γ treatments

Interferon-α enrichment analysis					
DeltaNeTS+	*GO terms*	Log2FC	*Reactome pathways*	DeMAND	*GO terms*
	Type I interferon signaling pathway		Interferon alpha/beta signaling		Defense response to virus
	Negative regulation of viral genome replication		Interferon Signaling_Homo sapiens		Type I interferon signaling pathway
	Negative regulation of viral life cycle		Cytokine Signaling in Immune system		Cellular response to type I interferon
	Cellular response to type I interferon		Immune System_Homo sapiens		Interferon-gamma-mediated signaling pathway
	Regulation of viral genome replication		Antiviral mechanism by IFN-stimulated genes		Response to type I interferon
	*Reactome pathways*		ISG15 antiviral mechanism		*Reactome pathways*
	Interferon signaling		Interferon gamma signaling		Interferon alpha/beta signaling
	Interferon alpha/beta signaling		RIG-I/MDA5 mediated induction of IFN-alpha/beta pathways		Interferon gamma signaling
			TRAF3-dependent IRF activation pathway		Interferon signaling
			Regulation of IFNA signaling		
			TRAF6 mediated IRF7 activation		
			Negative regulators of RIG-I/MDA5 signaling		
**Interferon-γ enrichment analysis**					
DeltaNeTS+	*GO terms*	Log2FC	*GO terms*	DeMAND	No enriched term
	Antigen processing and presentation of exogenous peptide antigen via MHC class II		Interferon-gamma-mediated signaling pathway		
			Cellular response to interferon-gamma		
	Antigen processing and presentation of exogenous peptide antigen		Cytokine-mediated signaling pathway		
			*Reactome pathways*		
	Antigen processing and presentation of peptide antigen via MHC class II		Interferon signaling		
			Interferon gamma signaling		
	Cellular response to interferon-gamma		Cytokine signaling in immune system		
	Interferon-gamma-mediated signaling pathway		Immune system		
	*Reactome pathways*		Interferon alpha/beta signaling		
	Interferon signaling		Translocation of ZAP-70 to immunological synapse		
	Interferon gamma signaling		Phosphorylation of CD3 and TCR zeta chains		
	MHC class II antigen presentation		PD-1 signaling		
	Cytokine signaling in immune system		MHC class II antigen presentation		
			Generation of second messenger molecules		

### Network perturbation analysis during influenza viral infections

Finally, we applied DeltaNeTS+ to analyze gene expression data from H1N1 and H5N1 influenza virus infected Calu-3 cells with the goal of elucidating the similarities and differences between the two important influenza viral strains. H1N1 strain is the cause of the 2009 swine flu outbreak and is known for its high transmissibility among humans. On the other hand, H5N1 strain is an avian influenza subtype that is known for its severe virulence with a high mortality rate of 60%. This high pathogenicity results in growing attention to understand the causal molecular mechanism [43]. Following DeltaNeTS+ analysis, we performed a GSEA to find over-represented Reactome pathways [37] and WGCNA [38] analysis to identify gene modules with similar dynamic perturbations, using the gene perturbation scores produced by DeltaNeTS+.

**Fig. 2:**
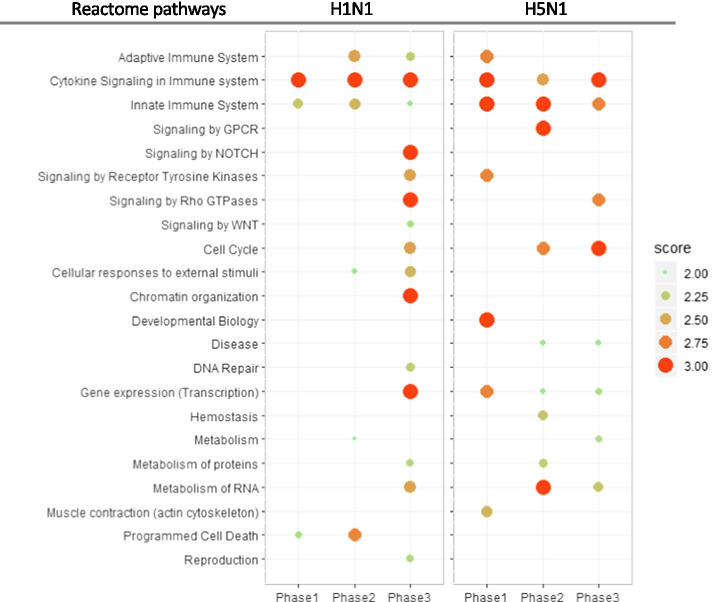
Summary of key Reactome pathways from gene set enrichment analysis of DeltaNeTS+ predictions. The size and color of dots indicate the score (negative logarithm-10 of *p* values) for the enriched Reactome terms. The influenza infection period is divided into three time phases: Phase1 = 0–7 h, phase 2 = 7–18 h, and phase 3 ≥ 18 h post-infection

Figure 2 depicts the Reactome pathways enriched (adjusted *p* value < 0.01, see Methods) across the three phases of the infection: early (phase 1: 0–7 h), middle (phase 2: 7–18 h) and late (phase 3: > 18 h) (full results in Additional file 4). H1N1 and H5N1 trigger many of the same cellular pathways but often in different phases. Expectedly, immune response, such as cytokine signaling and innate immune system, is triggered by both viral infections with roughly the same trend over time. Other pathways however are modulated with different timings. Among the pathways significantly enriched only in H5N1 infection are those associated with G-protein coupled receptor (GPCR) signaling and actin cytoskeleton (muscle contraction), both of which are known to be hijacked for viral entry processes (Fig. 2) [18, 44, 45]. On the other hand, programmed cell death and DNA damage (chromatin organization and DNA repair) are significantly enriched only in H1N1 data from the early through mid-phase of the infection. A previous study reported that death signaling molecules are downregulated in Calu-3 cells infected by H5N1 [46], a finding that is consistent with the absence of enrichment for programmed cell death in the result of DeltaNeTS+ analysis for H5N1. Other pathways enriched in the late phase of H1N1 infection are NOTCH, WNT, and RHO GTPase signaling, pathways that are relevant to cellular proliferation [47, 48]. These pathways are related to Histone cluster 1 families, and based on DeltaNeTS+ perturbation scores, are predicted to have been negatively perturbed (see Additional file 5). The negative perturbations signify a repression of cellular proliferation in H1N1 infection.

**Fig. 3:**
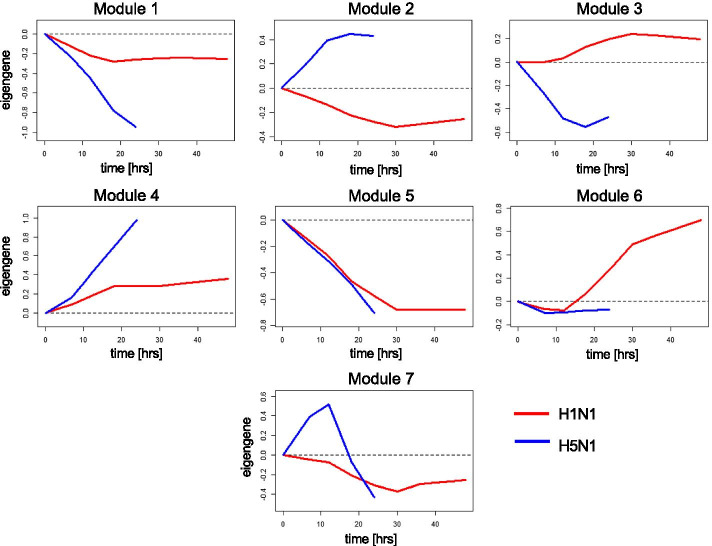
Eigengene profiles from WGCNA applied to DeltaNeTS+ perturbation scores of H1N1 and H5N1 influenza A infections (red: H1N1, blue: H5N1)

In addition to enrichment analysis, we applied WGCNA to the DeltaNeTS+ gene perturbation predictions to group the genes based on the pattern of perturbations over time. In this case, WGCNA finds clusters of genes whose perturbation scores given by the **P** matrix are highly similar based on a topological overlap derived from the gene–gene correlations. In the WGCNA results, the time-course perturbation values in each group can be represented by the first principal component in that group, called eigengene. Figure 3 shows the eigengene perturbation profiles of seven groups identified by WGCNA, and Table 3 provides the summary of GO and Reactome pathways enriched in each group (see details in Additional files 6 and 7). For the smallest group M7, no pathway was found to be significantly enriched.

**Table 3:** WGCNA modules of DeltaNeTS+ perturbation scores

Module	Size^a^	Summary of enriched pathways^c^	DB^d^
M1	2863	DNA replication, cell cycle	GO & RP
M2	2803	Influenza Viral RNA Transcription and Replication, viral mRNA Translation	GO & RP
M3	2645	Defense response to virus, immune response, interferon signaling	GO & RP
M4	1427	Viral entry (GPCR binding, amine transport)	GO & RP
M5	888	Crosslinking of collagen fibrils	RP
M6	762	Apoptosis (TNFR2 non-canonical NF-kB pathway)	RP
M7	397	N/A	–

*RP* reactome pathway database, *GO* gene ontology

^a^The number of genes clustered in each module

^b^Summary of enriched pathways (*q*-value < 0.10) in each module

^c^Pathway databases used for enrichment analysis

Groups M1, M4, and M5 are related to cell cycle arrest, viral entry, and cellular matrix barrier respectively. For these groups, the signs of the perturbations are the same for H1N1 and H5N1, but the magnitudes of the perturbations are larger for H5N1. For both strains, the infection acts to repress cell cycle arrest and cellular matrix barrier (negative perturbations) and to activate viral entry pathways (positive perturbations). For groups M2 and M3, the signs of the perturbations for the two influenza A strains are the opposite of each other. In H5N1 infection, viral RNA transcription, translation and replication (group M2) are induced, whereas viral defense and immune mechanisms (group M3) are strongly inhibited. In contrast, in H1N1 infection, viral RNA replication processes are repressed, but genes for antiviral mechanisms are moderately induced. Taken together, GSEA and WGCNA of DeltaNeTS+ for H1N1 and H5N1 infection in Calu3 indicates that in comparison to H1N1, the more virulent H5N1 infection shows increased activity of viral entry process and increased repression of cell cycle arrest, as well as inhibition of the immune system and programmed cell death.

## Discussion

DeltaNeTS+ is a network perturbation analysis tool for the analysis of gene expression data, which generates predictions on the direct gene expression perturbation in a given treatment or sample. DeltaNeTS+ builds on our previous algorithm DeltaNeTS which is able to utilize time-series datasets of gene expression, and adds the capability to take advantage of information on the GRN structure. The incorporation of GRN structure information enables accurate gene target predictions from a small number of samples, an advantage over our previous methods DeltaNet and DeltaNeTS. The direct gene perturbation of a gene is computed based on the difference between the measured differential expression of the gene and the differential expression predicted by the GRN model based on the expression of its transcription factors. We compared DeltaNeTS+ to three strategies, including (1) differential expression analysis, (2) a target inference method TSNI which relies on a model of the GRN [21], and (3) a network perturbation analysis method DeMAND which relies on statistical hypothesis testing via Kullback–Leibler divergence [9]. The comparative methods were selected since they are among the most commonly used and well-cited methods for gene target inference and are able to handle time-series data. DeltaNeTS+ is most similar to TSNI [21], as both methods rely on a linear regression formulation and time-series data. While TSNI uses principal component analysis (PCA) to reduce the dimension of the unknown GRN matrix **A** and perturbation matrix **P**, DeltaNeTS+ employs a regularization strategy (ridge regression or LASSO, depending on the availability of the GRN structure). TSNI is unable to accommodate information on GRN structure.

Adding GRN structural information to DeltaNeTS+ is highly beneficial and improves the gene target predictions, especially when the dataset contains only a few samples. The application of DeltaNeTS+ and a previous algorithm DeltaNet to *C. elegans*, T cell, and Calu-3 datasets without incorporating the GRN structure returned a null gene target prediction (**P** is **0**; data not shown). Here, by using the available GRN structure, DeltaNeTS+ is implemented using ridge regression in which the dimensionality of the unknown GRN matrix **A** is reduced to known TF-gene regulations. As shown in *C. elegans* and human T-cell case study, DeltaNeTS+ was able to use the GRN structure to predict genetic perturbations with high accuracy more reliably than the comparative methods. Besides, gene targets highly ranked by DeltaNeTS+ were closely associated with the true gene targets – for example, genes that are directly regulated by the true gene targets (see Additional file 1). The ability to utilize GRN structure information is important high-quality cell type specific gene regulatory networks become more available in recent times, thanks to the success of large-scale projects such as ENCODE [17] and FANTOM5 [49]. Besides, advanced network inference tools such as ARACNE [50] and MINDy [51] allow us to reverse-engineer a context-specific network from the gene expression data for a cell of our interest.

Note that DeltaNeTS+ is able to combine time-series and steady-state transcriptomic datasets for improving the accuracy of gene target predictions. For example, when we added a steady-state transcriptomic dataset (GSE51162) to the training of *C. elegans* GRN model in DeltaNeTS+, we were able to improve the rank of the predicted gene targets for *pie*-1 mutation (see Additional file 2: Table S3). Vice versa, the gene target predictions for the *C. elegans* steady-state dataset were better upon using the GRN model from the combination of time-series and steady-state data than using that from steady-state data alone (see Additional file 2: Figure S1). While incorporating the GRN structure information enables DeltaNeTS+ to predict gene targets using a small set of samples, the result above demonstrates that a larger number of samples improves accuracy. The capability of DeltaNeTS+ to integrate both time-series and steady-state data seamlessly in the gene target prediction means that DeltaNeTS+ will be able to take advantage of a larger library of available transcriptomic datasets and avoid running separate analyses for each kind of datasets.

The application of DeltaNeTS+ to the analysis of gene perturbation targets associated with H1N1 and H5N1 infection led to insights on the differences in the mechanism of actions between the two influenza A strains. In general, the more pathogenic strain H5N1 induces stronger and swifter perturbations than the less pathogenic but more infectious H1N1 (Figs. 2, 3). Notably, H5N1 infection shows a strong induction of viral entry process and viral replication and a repression of cell cycle arrest, all of which are strategies that would ensure a successful proliferation of the virus. The pathogenic H5N1 infection also demonstrates inhibition of viral defense mechanism. Furthermore, the less severity of H1N1 infection is also associated with a successful activation of cell death and repression of cell proliferation, pathways that are crucial in curtailing viral progression.

## Conclusions

DeltaNeTS+ is an effective network analysis method for inferring gene transcriptional perturbations from gene expression dataset. The ability of DeltaNeTS+ to integrate both steady-state and time-course gene expression data and available information of gene regulatory network structure enables accurate prediction of gene targets from limited number of samples (~ 10 s). The application of DeltaNeTS+ to influenza A infection datasets in human Calu-3 give insights into the key functional perturbations that differ between highly virulent H5N1 avian flu and highly transmissible H1N1 swine flu. Insights on the molecular mechanisms of drugs and diseases from gene target predictions provide important information for drug discovery and treatment formulations. The ability to marry gene expression profiles with transcriptional network structures derived from genomic information, as done in DeltaNeTS+, is crucial for understanding diseases and for formulating individualized treatments in the era of personalized medicine.

## Supplementary Information

**Additional file 1**. Genes with higher ranking than the true targets in Caenorhabditis elegans dataset

**Additional file 2: Table S1**. Gene target ranking by DeltaNeTS+ and log2FC analysis for each time point in C. elegans datasets. Table S2. Gene target ranking by DeltaNeTS+ and log2FC analysis for each time point in STAT6 siRNA experiments of human T-cell data. Table S3. Gene target prediction of DeltaNeTS+ for time-series C. elegans data (GSE2180) upon using time-series data alone and using a combination of time-series and steady-state (GSE51152) dataset. Figure S1. Gene target ranking by DeltaNeTS+ for steady-state C. elegans data (GSE51162) upon using the GRN model learned on steady-state dataset alone and using the GRN model trained on steady-state and time-series (GSE2180) datasets (*p value < 0.05 by Wilcox signed rank test)

**Additional file 3**. Enriched GO terms and Reactome pathways of the top gene target predictions from DeltaNeTS+, DeMAND and log2FC analysis

**Additional file 4**. Reactome pathway enrichment analysis across the three phases of H1N1 and H5N1 influenza-A infection in Calu-3: early (phase 1: 0-7hr), middle (phase 2: 7-18hrs) and late (phase 3: >18hrs)

**Additional file 5**. DeltaNeTS+ analysis of H1N1 and H5N1 influenza-A infection in Calu-3: early (phase 1: 0-7hr), middle (phase 2: 7-18hrs) and late (phase 3: >18hrs)

**Additional file 6**. GO enrichment analysis of WGCNA modules derived from DeltaNeTS+ analysis of H1N1 and H5N1 influenza-A infection in Calu-3 (terms with adjusted p-value < 0.5 are highlighted in red)

**Additional file 7**. Reactome pathway enrichment analysis of WGCNA modules derived from DeltaNeTS+ analysis of influenza-A infection in Calu-3 (pathway with q-value < 0.1 are highlighted in red)

## Data Availability

Project name: DeltaNeTS+. Project home page: http://www.github.com/cabsel/deltanetsplus. Operating system(s): Platform independent. Programming language: R. Other requirements: R 3.6.3 or higher. License: GNU GPL. Any restrictions to use by non-academics: Licence needed.

## Abbreviations

ARACNE: Algorithm for the Reconstruction of Accurate Cellular Networks
CMap: Connectivity Map
DeMAND: Detecting Mechanism of Action based on Network Dysregulation
ENCODE: ENcyclopedia of DNA Elements
FANTOM5: Functional ANnoTation of the Mammalian genome
GAS: Gamma interferon Activation Sites
GO: Gene Ontology
GPCR: G-Protein Coupled Receptor
GRN: Gene Regulatory Network
GSEA: Gene Set Enrichment Analysis
IFN-α: Interferon-α
IFN-γ: Interferon-γ
ISG: IFN-α/β Stimulated Genes
LASSO: Least Absolute Shrinkage and Selection Operator
Log2FC: Log-2 Fold Change
MHC: Major Histocompatibility Complex
MINDy: Modulatory Inference by Network Dynamics
MNI: Mode of action by Network Identification
MOA: Mechanism of Action
NIR: Network Identification by multiple Regression
ODE: Ordinary Differential Equation
PCA: Principal Component Analysis
RMA: Robust Multi-array Average
RNAi: RNA interference
SSEM: Sparse Simultaneous Equation Model
TF: Transcription Factor
TSNI: Time Series Network Identification
WGCNA: Weighted Gene Co-expression Network Analysis
