# Supplementary material for: DeltaNeTS+: elucidating the mechanism of drugs and diseases using gene expression and transcriptional regulatory networks
Source: BMC Bioinformatics. 2021 Mar 4;22:108. doi: 10.1186/s12859-021-04046-2 (PMC7934467; doi:10.1186/s12859-021-04046-2)
Supplement: Supplementary file 2 — Additional file 2: Table S1. Gene target ranking by DeltaNeTS+ and log2FC analysis for each time point in C. elegans datasets. Table S2. Gene target ranking by DeltaNeTS+ and log2FC analysis for each time point in STAT6 siRNA experiments of human T-cell data. Table S3. Gene target prediction of DeltaNeTS+ for time-series C. elegans data (GSE2180) upon using time-series data alone and using a combination of time-series and steady-state (GSE51152) dataset. Figure S1. Gene target ranking by DeltaNeTS+ for steady-state C. elegans data (GSE51162) upon using the GRN model learned on steady-state dataset alone and using the GRN model trained on steady-state and time-series (GSE2180) datasets (*p value < 0.05 by Wilcox signed rank test) [file 12859_2021_4046_MOESM2_ESM.docx]

**Supplementary Table S1.** Gene target ranking by DeltaNeTS+ and log2FC analysis for each time point in *C. elegans* datasets

| EXP1: skn-1 shRNA on mex-3 mutated cells | | | | |
| --- | --- | --- | --- | --- |
| Time [min] | mex-3 repression | | skn-1 silencing | |
|  | DeltaNeTS+ | log2FC | DeltaNeTS+ | log2FC |
| 0 | 3 | 1 | 39 | 911 |
| 23 | 4 | 2 | 46 | 6242 |
| 41 | 2 | 2 | 61 | 3889 |
| 53 | 11 | 14 | 58 | 16010 |
| 66 | 5 | 15 | 93 | 12632 |
| 83 | 7 | 137 | 210 | 13133 |
| 101 | 5 | 403 | 419 | 9266 |
| 122 | 7 | 660 | 381 | 8405 |
| 143 | 4 | 1059 | 487 | 8181 |
| 186 | 238 | 2720 | 487 | 6575 |
| EXP2: pal-1 shRNA on pie-1 mutated cells | | | | |
| Time [min] | pie-1 repression | | pal-1 silencing | |
|  | DeltaNeTS+ | log2FC | DeltaNeTS+ | log2FC |
| 0 | 2 | 1 | 1 | 2 |
| 23 | 2 | 1 | 1 | 4 |
| 41 | 4 | 2 | 1 | 1555 |
| 53 | 4 | 6 | 1 | 391 |
| 66 | 5 | 20 | 1 | 2009 |
| 83 | 8 | 32 | 1 | 195 |
| 101 | 8 | 55 | 1 | 10411 |
| 122 | 41 | 215 | 1 | 293 |
| 143 | 291 | 1787 | 2 | 9270 |
| 186 | 1029 | 2927 | 1 | 1565 |
| EXP3: pie-1 mutation | | | | |
| Time [min] | pie-1 repression | | | |
|  | DeltaNeTS+ | | log2FC | |
| 0 | 1 | | 1 | |
| 23 | 1 | | 1 | |
| 41 | 1 | | 1 | |
| 53 | 1 | | 1 | |
| 66 | 1 | | 25 | |
| 83 | 1 | | 29 | |
| 101 | 2 | | 122 | |
| 122 | 1 | | 311 | |
| 143 | 1 | | 1335 | |
| 186 | 7 | | 3385 | |

**Supplementary Table S2.** Gene target ranking by DeltaNeTS+ and log2FC analysis for each time point in STAT6 siRNA experiments of human T-cell data

|  | EXP1: STAT6 siRNA | | EXP2: STAT6 siRNA + with IL-4 | |
| --- | --- | --- | --- | --- |
| Time [h] | STAT6 silencing | | STAT6 silencing | |
|  | DeltaNeTS+ | log2FC | DeltaNeTS+ | log2FC |
| 0 | 1 | 2 | - | - |
| 12 | 1 | 1 | 1 | 2 |
| 24 | 1 | 3 | 1 | 12 |
| 48 | 1 | 2 | 2 | 92 |
| 72 | 1 | 13 | 3 | 152 |

**Supplementary Table S3.** Gene target prediction of DeltaNeTS+ for time-series *C. elegans* data (GSE2180) upon using time-series data alone and using a combination of time-series and steady-state (GSE51152) dataset.

|  | **Rank of Known Gene Targets by DeltaNeTS+** | | | | |
| --- | --- | --- | --- | --- | --- |
|  | Experiment 1 | | Experiment 2 | | Experiment 3 |
| **Training Data** | *mex*-3 | *skn*-1 | *pie*-1 | *pal*-1 | *pie*-1 |
| Time-series alone | 9 | 44 | 9 | 2 | 2 |
| Time-series + Steady-state | 9 | 55 | 1 | 2 | 1 |


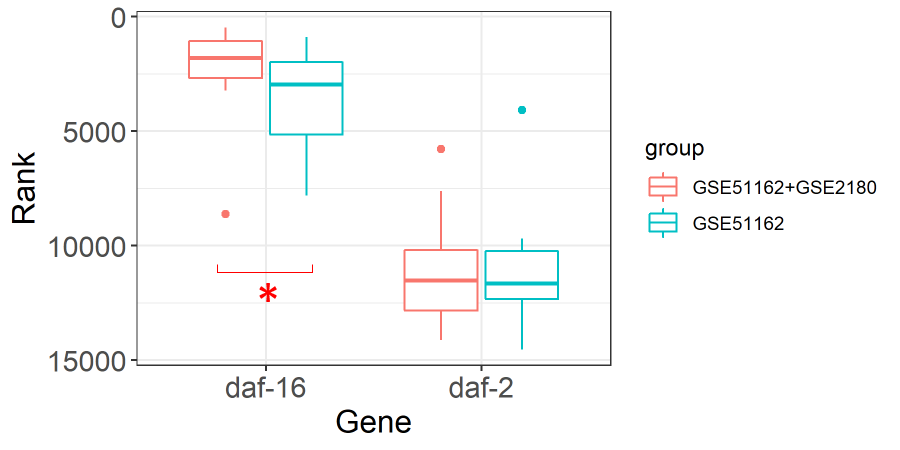


**Supplementary Figure S1.** Gene target ranking by DeltaNeTS+ for steady-state *C. elegans* data (GSE51162) upon using the GRN model learned on steady-state dataset alone and using the GRN model trained on steady-state and time-series (GSE2180) datasets. *: *p*-value < 0.05 by Wilcox signed rank test.
